# The Effect of Rearing Conditions on Carcass Traits, Meat Quality and the Compositions of Fatty Acid and Amino Acid of LTL in Heigai Pigs

**DOI:** 10.3390/ani12010014

**Published:** 2021-12-22

**Authors:** Jie Li, Jiaqi Liu, Shu Zhang, Jintang Xie, Tizhong Shan

**Affiliations:** 1 College of Animal Sciences, Zhejiang University, Hangzhou 310058, China; 21917077@zju.edu.cn (J.L.); liu-jiaqi@zju.edu.cn (J.L.); 22017056@zju.edu.cn (S.Z.); 2 The Key Laboratory of Molecular Animal Nutrition, Zhejiang University, Ministry of Education, Hangzhou 310058, China; 3 Key Laboratory of Animal Feed and Nutrition of Zhejiang Province, Hangzhou 310058, China; 4 Shandong Chunteng Food Co., Ltd., Zaozhuang 277500, China; sdctxjt@163.com

**Keywords:** pig, rearing condition, meat quality, fatty acid composition

## Abstract

**Simple Summary:**

People’s demand for meat consumption has transformed from quantity to quality. The rearing condition is one of the factors affecting meat quality. However, the effects of different rearing conditions on the production of Chinese indigenous pig breeds are still barely understood. In this study, Heigai pigs (a Chinese indigenous pig breed) were raised in the indoor feeding farm and the grazing farm to investigate the effects of different rearing conditions on carcass traits, meat quality and the compositions of fatty acid and amino acid. Grazing farm pigs tended to increase shear force, while significantly increasing the saturated fatty acid ratio and decreasing the unsaturated fatty acid ratio to alter the composition of fatty acids of longissimus thoracis et lumborum. The present study provides an experimental reference for regulating the production of superior meat quality pork of Chinese local breed pigs.

**Abstract:**

The present study evaluates the influence of captivity and grazing rearing conditions on the carcass traits, meat quality and fatty acid profiles of Heigai pigs. Ten Heigai pigs with market weight were randomly selected from both the indoor feeding farm and outdoor grazing farm groups (FF and GF; five pigs per group) for measuring production performance. The results showed that the shear force of longissimus thoracis et lumborum (LTL) in the GF group tended to increase (*p* = 0.06), and triglyceride and cholesterol contents in LTL and psoas major muscle (PMM) of the GF group significantly increased and decreased, respectively (*p* < 0.05). The proportion of saturated fatty acids (SFA) was significantly increased (*p* < 0.05) in the GF group. Meanwhile, the ratios of unsaturated fatty acid (UFA), polyunsaturated fatty acid (PUFA), monounsaturated fatty acid (MUFA) and the content of flavor amino acid of the LTL in the GF group were significantly decreased (*p* < 0.05). The GF upregulated the expression of *MyHC-IIb* and lipogenic genes, such as *GLUT4* and *LPL* (*p* < 0.05), in LTL and PMM, but downregulated the expression of *MyHC-I,* *MyHC-IIa*, *PPARγ* and *leptin* (*p* < 0.05). In conclusion, these results suggested that the different rearing conditions can alter the meat qualities by mediating the muscle fiber type and lipid metabolism of Heigai pigs.

## 1. Introduction

Meat quality assessment is a comprehensive concept, including meat appearance, color, tenderness, juiciness and flavor, which largely depends on the breed, nutrition, rearing system and post-slaughter handling [1]. In addition to the above external factors, muscle fiber types, proportion and lipid composition of intramuscular fat (IMF) also play an important role in the determination of meat quality [2]. In general, muscle fibers are generally classified into four types: I, IIa, IIx and IIb. Type I fibers (slow muscle fibers) have greater oxidative capacity to support sustained muscle contractions that exhibit low-intensity contractions, whereas type II fibers (fast muscle fibers) are predominantly glycolytic fibers that utilize the rapid conversion of glycogen for short bursts of energy [3]. The glycolytic and oxidative capacities are also closely associated with metabolic enzymes, including lactate dehydrogenase (LDH), succinic dehydrogenase (SDH) and malate dehydrogenase (MDH) [4]. In addition, the composition of muscle fibers is also related to IMF deposition [5], as type I and IIa fibers have large amounts of lipid accumulation, whereas IIx and IIb fibers allow less storage of lipids [6]. The number and composition of different types of muscle fibers directly affect various aspects of meat quality and are critical to its nutritional value. Furthermore, the effect of housing (indoor) and grazing (outdoor/free-range) rearing conditions on meat quality has also been widely explored and discussed.

The fatty acid profile is a crucial index of meat quality, and adequate proportions of saturated fatty acids (SFA), monounsaturated fatty acids (MUFA) and polyunsaturated fatty acids (PUFA) can ensure the excellent taste and nutritional quality of meat [7]. In addition, polyunsaturated fatty acids (PUFA), especially the n–3 fatty acids, are considered to be functional components that affect the health of animals and humans [8]. The content of flavor amino acids, such as glycine, alanine, aspartic acid, glutamic acid, phenylalanine, and tyrosine in meat is an important factor affecting the sensory quality of meat and directly affects its flavor features [9]. Consequently, continuously providing high quality pork with better muscle fiber characteristics, higher content of IMF, beneficial fatty acid and amino acid compositions is urgently required by today’s pig production industry.

Chinese local pig breeds are considered as an important source of breeds with better IMF deposition and meat quality than crossbred pigs [6,10]. Intensive selective breeding and genetic improvement of crossbred pigs led to the abandonment of many low productive local pig breeds, and the deterioration in the quality of pork [11]. Heigai pig is a fatty breed in northern China renowned for its meat quality, reproductive performance, adaptability and disease resistance [12,13]. At present, there are few studies on Heigai pigs, and there is no report of research on the effect of rearing conditions on the meat quality of Heigai pigs. In this study, Heigai pigs were used as experimental animals to compare the carcass traits, meat quality, muscle fiber characteristics and metabolic enzyme activities of different rearing conditions. Moreover, we examined the fatty acid and amino acid profiles between the indoor housing and grazing rearing conditions of Heigai pigs to clarify the impact of rearing conditions on meat quality traits, and provided a reference for the selection of rearing conditions in Chinese local swine productions.

## 2. Material and Methods

### 2.1. Animals

Animal studies were approved by the Institutional Animal Care and Use Committee of Zhejiang University (Zhejiang, China). The ethical committee number for the study is ZJU20170466. The present study was carried out in the Shandong Chunteng Food Co., Ltd. (Shandong, China). A total of 60 Heigai pigs were randomly allocated to 2 groups with 5 replications (6 pigs per replication) and raised in the indoor feeding farm (FF) and free grazing farm (GF). In the fattening stage, the pigs in two groups were fed the same general compound diet and free intake. Then one pig ( reached the market weight and close to the average body weight) per replication was selected from each group for further study.

### 2.2. Sample Collection, Carcass Characteristics and Meat Quality Measurements

All pigs were fasted before sampling. After fasting overnight, the carcass indexes and meat quality were determined. The longissimus thoracis et lumborum (LTL) and psoas major muscle (PMM) were quickly collected, frozen in liquid nitrogen and stored at −80 degrees Celsius. The samples were used for the subsequent determination of fatty acid composition, amino acid profile, intramuscular fat (IMF) content, enzyme activity and gene expression.

The cross-sectional area of LTL (height × width × 0.7) at the junction of the 6th to 7th rib was measured as the loin muscle area (cm^2^). Backfat thickness (mm) was calculated by averaging the scores of three regions at the first rib, last rib and last lumbar vertebrae of the right carcass sides. A portable pH meter (pH-Star Matthäus GmbH, Pöttmes, Germany) was used to measure the pH values of the LTL stored at 4 °C for 1 h (pH1) and 24 h (pH24) after slaughter. The sample was cut into about 10 g each, hung in a special sealed plastic tube and weighed at 4 °C after 24 h. We calculated the percentage of weight loss to initial weight as drip loss (%). Backfat thickness, loin area and meat quality traits were measured three times. The shear force and the IMF content were measured according to previously published method [14].

### 2.3. Triglyceride and Cholesterol Contents Measurement

The samples of LTL and PMM were homogenized according to the tissue weight and 0.86% of the normal saline solution volume ratio of 1:9, centrifuged for 10 min, and the supernatant was taken into a new centrifugal tube for subsequent determination. The triglyceride and cholesterol contents of LTL and PMM were measured using the corresponding assay kits (TG, A110-1-1; TC, A111-1-1) produced by the Nanjing Jiancheng Bioengineering Institute (Nanjing, China), according to the manufacturer’s instructions. At the same time, the total protein of the homogenate was determined using a BCA Protein Assay Reagent Kit (Thermo Fisher Scientific Inc., Waltham, MA, USA), as previously described [12].

### 2.4. Metabolic Enzyme Activities Measurement

The activities of LDH, SDH and MDH were measured using commercial kits (LDH, A020-2; SDH, A022; MDH, A021-2) produced by the Nanjing Jiancheng Institute of Bioengineering (Nanjing, China), according to the manufacturer’s instructions.

### 2.5. Analysis of the Fatty Acid Composition and Amino Acid Profile

The fatty acid profiles of the LTL samples were measured by gas chromatography, as previous study [15]. First, total lipids in the LTL samples were extracted by the chloroform–methanol (2:1, *v*/*v*) procedure. Then, a gas chromatography (GC; Model 7890A, Agilent Technologies Inc., Santa Clara, CA, USA) was used to determine the fatty acid methyl esters, which were converted from the total fat lipids through KOH/methanol. The concentration of a single fatty acid was expressed as a percentage of the total fatty acid, which was determined by the retention time of the peak area compared with a known standard (Sigma Chemical Co., St. Louis, MO, USA). The amino acid composition was determined by the public platform of the Zhejiang Provincial Laboratory of Feed and Animal Nutrition.

### 2.6. Total RNA Extraction and Quantitative Real-Time PCR

The Total RNA was extracted from muscle samples using TRIzol reagent (Thermo Fisher Scientific Inc., Waltham, MA, USA) and the qualified RNA samples, which were determined by a NanoDrop 2000 (Thermo Fisher) instrument, were used for subsequent reverse transcription. The cDNA was synthesized by reverse transcription from RNA using random primers and a ReverAid First Strand cDNA Synthesis Kit (Thermo Fisher). The protocols and specific primers used for qPCR were mentioned, as previously described [12].

### 2.7. Statistical Analysis

Statistical analysis was performed using GraphPad Prism 6 software, package by two-tailed Student’s *t*-tests using the SPSS 20.0 software (IBM-SPSS Inc., Chicago, IL, USA). All statistical data were expressed as the mean ± SEM. Statistical significance was established at *p* < 0.05. On the basis of the Student’s *t*-tests, the power analysis was performed to help determine the effect size (Cohen’s d) between the groups assuming at least 80% power, to detect the effects of the particular sample size from baseline to post-test at the 0.05 level of the *p* value, and the effect size (>2.0) of the present study was considered as a large size effect, the magnitude of which was considered reasonable for the feasibility study [16,17].

## 3. Results

### 3.1. Effects of Rearing Condition on Carcass Traits and Meat Quality

The carcass traits and meat quality of Heigai pigs in the GF group and FF group were presented in Table 1. The results showed that the rearing conditions had no significant effect on the carcass traits, including slaughter weight, carcass weight, lean percentage, backfat thickness and loin muscle area. Moreover, the Heigai pigs performed similar characteristics of meat quality, including pH1, pH24, shear force, intramuscular fat (IMF) content and drip loss between the GF and FF groups. Notably, the shear force of LTL in the GF group was higher than the FF group (*p* = 0.06), suggesting that rearing conditions can mainly affect the shear force of LTL in Heigai pigs.

### 3.2. Effects of Rearing Conditions on Fatty Acid Profiles in LTL

To investigate the differences in fatty acid compositions between the FF and GF groups, the overall fatty acid profiles were measured in LTL (Table 2) of Heigai pigs.

As shown in Table 2, the contents of C10:0 (*p* < 0.01), C20:0 (*p* < 0.01), C22:0 (*p* < 0.05), were significantly lower in the GF group contrasted to the FF group. However, there were higher contents of C12:0 and C16:0 (*p* < 0.01) in the GF group as well as the sum of saturated fatty acids (SFAs). In contrast, the contents of the total unsaturated fatty acids (UFAs), including monounsaturated fatty acid (MUFA) and polyunsaturated fatty acid (PUFA), in the GF group decreased significantly. Moreover, the contents of palmitic, C18:1 n-9, C18:2 n-6, C20:1 and C20:3 (*p* < 0.01) were significantly lower in the GF group and the contents of C20:4 n-6, C22:1 and C22:6 were also significantly lower in GF group than the FF group (*p* < 0.05). The ratio of n-6/n-3 PUFA significantly decreased in the GF group (*p* < 0.01).

### 3.3. Effects of Rearing Conditions on Free Amino Acid Profiles in LTL

Amino acid composition is also an important factor in determining meat quality. According to the amino acid profiles in LTL of Heigai pigs, it was found that the total protein of the FF group was analogous to that of the GF group (Table 3). Interestingly, the contents of serine, proline, glycine, alanine, methionine, phenylalanine and histidine were significantly higher in the GF group than the FF group (*p* < 0.05), and the contents of aspartate and threonine were significantly lower in the GF group than the FF group (*p* < 0.05).

### 3.4. Effects of Rearing Conditions on Muscle Fiber Type Composition and Metabolic Enzyme Activity

**Figure 1:**
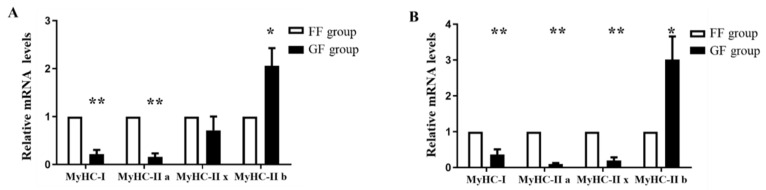
The expression of myosin heavy chain (MyHC) isoforms in the longissimus thoracis et lumborum (LTL) and psoas major muscle (PMM) of Heigai pigs. (**A**) Relative mRNA expression levels of MyHC isoforms in LTL. (**B**) Relative mRNA expression levels of MyHC isoforms in PMM. Each column represents the means ± SEM (*n* = 5). * *p* < 0.05; ** *p* < 0.01.

We examined the expression levels of muscle fiber type in LTL and PMM, on account of muscle fiber type composition closely related to shear force. A significant increase in glycolytic fiber type (type IIb) expression level was observed in the LTL and PMM of the GF group compared with the FF group (*p* < 0.05, Figure 1A,B), while the oxidative fiber types (types I, IIa) were expressed at a significantly low level in the GF group than the FF group in LTL and PMM (*p* < 0.01, Figure 1A,B).

Moreover, we measured the metabolic enzyme activities of LDH, SDH and MDH in the LTL and PMM of Heigai pigs (Table 4). The results showed that the oxidative enzyme activities of MDH and SDH were lower in the GF group than the FF group in LTL (*p* < 0.05). However, compared to the FF group, the LDH activity was significantly higher in the PMM of the GF group (*p* < 0.05), which reflected the high glycolytic capacities. The results above demonstrated that Heigai pigs exhibited higher glycolytic capacities under the grazing system.

### 3.5. Effects of Rearing Conditions on mRNA Expression Levels of Lipid Metabolism

**Figure 2:**
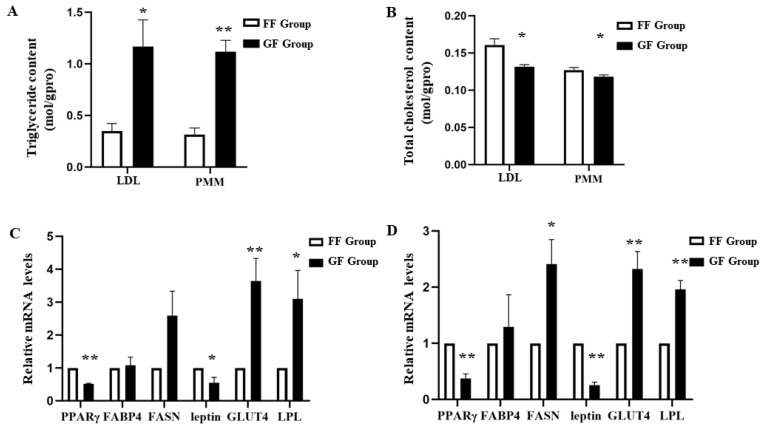
The expression of CRTC3 and adipogenesis related genes in the longissimus thoracis et lumborum (LTL) and psoas major muscle (PMM)of Heigai pigs. (**A**) Triglyceride contents in the LTL and PMM. (**B**) Total cholesterol contents in the LTL and PMM. (**C**) Relative mRNA expression levels of adipogenesis related genes, including PPARγ, FABP4, FASN, *leptin*, GLUT4 and LPL in LTL. (**D**) Relative mRNA expression levels of adipogenesis related genes in PMM. Each column represents the means ± SEM (*n* = 5). * *p* < 0.05; ** *p* < 0.01.

The results above showed that IMF contents were similar in the GF and the FF groups of Heigai pigs. Interestingly, it was found that the triglyceride contents of the GF group were significantly higher than those of the FF group in LTL (*p* < 0.05) and PMM (*p* < 0.01; Figure 2A). The total cholesterol contents in the LTL and PMM of the GF group were significantly lower than those of the FF group (*p* < 0.05; Figure 2B). Therefore, Heigai pigs in the GF group produced healthier pork with a higher triglyceride content and a lower total cholesterol content than that of the FF group. To further investigate the mechanism of diverse lipid metabolism between the GF and FF groups, the mRNA expression levels of adipose deposition related genes were measured in the LTL and PMM of Heigai pigs (Figure 2C,D). Compared to the FF group, the mRNA expression level of glucose transporter 4 (*GLUT4*) was significantly higher in the GF group, both in LTL and PMM (*p* < 0.01). Consistently, the mRNA expression level of lipoprotein lipase (*LPL*) was significantly higher in the GF group, both in LTL (*p* < 0.05) and PMM (*p* < 0.01). Moreover, the mRNA expression level of fatty acid synthase (*FASN*) was significantly increased in the PMM of the GF group than the FF group (*p* < 0.01), while the expression levels of the peroxisome proliferator-activated receptor γ (*PPARγ*) and *leptin* were significantly decreased (*p* < 0.01). In contrast to the FF group, the expression level changes of *PPARγ* and *leptin* are consistent with the PMM in the LTL of the GF group (*p* < 0.01). However, there was no difference in the expression level of fatty acid binding protein 4 (*FABP4*) both in the LTL and PMM of the GF and FF groups.

## 4. Discussion

In the present study, we examined the effect of rearing conditions (indoor housing and grazing) on the carcass traits, meat quality, fatty acid profiles and amino acid composition of Heigai pigs. We found that GF Heigai pigs performed more glycolytic myofibers and high metabolic enzyme activities, which can partly explain the larger value of shear force.

Previous studies did not find significant differences in carcass traits and meat quality characteristics between the enclosed and grazing rearing conditions [18,19]. Our study showed similar results to the above mentioned, except for the different value of the shear force between the FF and GF groups (*p* = 0.06). These results indicated that the grazing farm system was positively associated with the shear force of Heigai pigs, in contrast to feeding farm conditions. Moreover, we found that Heigai pigs in the GF group had a higher expression of glycolytic muscle fibers (type IIb), a lower expression of oxidative muscle fibers (type I and IIa) than that in the FF group, in both the LTL and PMM. Thus, in comparison with the FF group, Heigai pigs in the GF group can perform a shift of muscle fibers from the oxidative muscle fiber type (I and IIa) to the glycolytic muscle fiber type (Iix), compared to feeding pigs. Consistently, it was suggested that grazing pigs had a higher expression level of MyHC IIb and MyHC IIx than feeding pigs [20]. Other studies, pointed out that spontaneous physical activity significantly increased the proportion of fiber types (IIb/x), rather than the content of the muscle fiber types (IIb) [21]. To further study the activities of metabolic enzymes in the grazing and feeding conditions of Heigai pigs, we examined the activities of glycolytic metabolism enzymes (LDH) and oxidative metabolism enzymes (MDH and SDH). Our results showed lower activities of MDH and SDH in LTL, and higher activities of LDH in PMM of pigs in the GF group than those in the FF group. Hamill et al. found a negative impact of the abundance of fast fibers and high glycolytic metabolism on meat tenderness [22]. However, some studies showed that grazing pigs had more oxidation and less glycolysis muscle metabolism [23]. The increased levels of type IIx fiber in the LTL and PMM of the GF Heigai pigs can be due to more physical activity.

A recent study showed that muscle fiber type composition and IMF content were unrelated to the effects of the breeding system [23]. Indeed, similar IMF contents were found in the GF and FF groups. In addition, we measured the triglyceride contents and total cholesterol in the LTL and PMM. Interestingly, higher triglyceride contents and lower cholesterol contents were found in the GF group, but not in the FF group of Heigai pigs. In general, lower cholesterol levels in pork can be more popular for consumers to prevent cardiovascular diseases [24]. Furthermore, we examined the expression levels of adipose deposition-related genes, including *PPARγ*, *FABP4*, *FASN*, *leptin*, *GLUT4* and *LPL* in muscle tissue of Heigai pigs. Lower expression levels of *PPARγ* and *leptin*, and higher expression levels of *GLUT4* and *LPL* were found in the GF group than those of the FF group, in the LTL and PMM. Consistent with previous studies, it was suggested that *PPARγ* and *leptin* were significantly downregulated after long-term exercise [25,26]. In the skeletal muscles, the *GLUT4* and *LPL* were increased in response to sprint interval exercise [27]. Triglyceride and cholesterol are used to supply energy in exercise. Therefore, adipose deposition related genes which can affect the accumulation and composition of fatty acids in muscle tissue were regulated in grazing Heigai pigs. 

The fatty acid composition of muscle tissue plays an important role in the regulation of adipose deposition that determines the oxidative stability of muscle and meat quality [28]. Here, we found that the fatty acid compositions in LTL were significantly changed under grazing raising conditions. The GF increased the level of SFA, and decreased the portion of PUFA and MUFA in LTL. Higher SFA is positively associated with the degree of fat firmness that directly affects the meat quality [29]. A higher ratio of SFA/UFA is positively correlated with oxidative prevention and maintains the pH value after slaughter. Previous studies demonstrated an increase in the PUFA portion in the adipose tissue of grazing pigs [30]. In addition, the majority of fatty acids in the LTL consist of oleic (C18:1), palmitic (C16:0) and stearic (C18:0) acids. Grazing affected the fatty acid composition with increasing palmitic acid and decreasing steric acid in LTL. It is reported that apple pomace-mixed silage can increase palmitic acid and decrease steric acid in backfat, which can be related to dietary fiber [31]. More importantly, the researches on functional pork related to fatty acid composition mainly focus on the n-6/n-3 PUFA ratio, and n-3 PUFA and n-6PUFA are closely related to the occurrence and development of human diseases. It is generally accepted that a lower ratio of n-6/n-3 PUFA in meat is beneficial for human health [13,32,33]. Moreover, a decreased ratio of n-6/n-3 PUFA was found in grazing pigs, which is consistent with the findings of others [30]. Thus, grazing pigs are more likely to provide foods containing functional ingredients, which can promote health and improve meat quality.

Grazing not only changed the fatty acid composition and fat quality of pigs, but also affected the amino acid composition [34]. The results showed that there was no difference in total protein contents between different rearing conditions. However, it was noteworthy that the flavor potentiating effects on amino acids, glycine, alanine and tyrosine were increased in grazing pigs. Better flavor that can be considered as high meat quality is popular for consumers. Taken together, our study compared the difference of carcass and meat quality traits between free grazing farm and indoor feeding farm conditions. We observed that the GF Heigai pigs had more glycolytic myofibers and metabolic enzyme activities, a higher triglyceride content and a lower cholesterol content in the muscle tissue. Combined with the profiles of fatty acid and amino acid composition, we suggested that GF Heigai pigs had a better meat quality and higher nutritional value than FF Heigai pigs. However, it should be noted that the relatively small sample size in the present study may not be representative, and further research is still needed to fully reveal the impact of the different rearing systems on the meat quality of pork.

## 5. Conclusions

In conclusion, the present study demonstrated that there were no differences in the carcass traits between the GF and FF groups, but GF conditions used in this study tended to regulate the shear force in LTL, which was possibly associated with the transformation of oxidative muscle fiber to glycolytic muscle fiber. Moreover, the GF changed the fatty acid composition in the meat, while containing similar intramuscular fat content compared to the FF, and had significantly lower cholesterol contents, which can be more beneficial to human health. Although the grazing/rearing condition did not influence the total protein content, it significantly altered the amino acid composition, which made the flavor amino acids increase in the GF pigs. In summary, these results suggest that the grazing/rearing condition altered the meat quality by possibly mediating the muscle fiber type and lipid metabolism of Heigai pigs.

## Figures and Tables

**Table 1:** Characteristics of carcass and meat quality traits under different rearing conditions in Heigai pigs.

Items	FF	GF
Slaughter weight (kg)	107.00 ± 3.05	108.50 ± 1.34
Carcass weight (kg)	79.88 ± 2.47	79.32 ± 1.24
Lean percentage (%)	50.25 ± 1.75	50.46 ± 0.58
Backfat thickness (mm)	36.57 ± 3.01	36.52 ± 2.74
Loin muscle area (cm^2^)	30.83 ± 1.92	31.54 ± 0.98
pH1	6.05 ± 0.20	6.27 ± 0.04
pH24	5.61 ± 0.03	5.66 ± 0.01
Shear force (N)	42.86 ± 2.19	55.77 ± 2.46
Intramuscular fat (%)	3.10 ± 0.04	3.10 ± 0.03
Drip loss (%)	1.60 ± 0.09	1.66 ± 0.02

Note: FF (indoor feeding farm) group, *n* = 5; and GF (free grazing farm) group, *n* = 5. SEM: standard error of the mean.

**Table 2:** Fatty acid composition of LTL(longissimus thoracis et lumborum ) under different rearing conditions in Heigai pigs.

FA (%)	FF	GF
C8:0	0.18 ± 0.01	0.19 ± 0.01
C10:0	0.06 ± 0.01 ^A^	0.05 ± 0.01 ^B^
C12:0	0.43 ± 0.01 ^B^	0.53 ± 0.01 ^A^
C14:0	2.28 ± 0.05	2.33 ± 0.04
C15:0	0.20 ± 0.01	0.23 ± 0.01
C16:0	29.97 ± 0.26 ^B^	31.35 ± 0.14 ^A^
C18:0	12.65 ± 0.06	13.20 ± 0.35
C20:0	0.63 ± 0.01 ^A^	0.53 ± 0.01 ^B^
C22:0	0.08 ± 0.01 ^a^	0.07 ± 0.01 ^b^
C24:0	0.02 ± 0.01	0.02 ± 0.01
∑ SFA	46.50 ± 0.17 ^B^	48.49 ± 0.28 ^A^
C16:1	2.38 ± 0.06 ^A^	2.12 ± 0.03 ^B^
C18:1 n-9	45.64 ± 0.19 ^A^	44.55 ± 0.25 ^B^
C18:2 n-6	3.43 ± 0.07 ^A^	2.92 ± 0.01 ^B^
C18:3 n-3	0.39 ± 0.01	0.41 ± 0.01
C20:1	0.77 ± 0.02 ^A^	0.70 ± 0.01 ^B^
C20:2	0.33 ± 0.01	0.32 ± 0.01
C20:3	0.15 ± 0.01 ^A^	0.13 ± 0.01 ^B^
C20:4 n-6	0.31 ± 0.01 ^a^	0.27 ± 0.01 ^b^
C20:5	0.02 ± 0.01	0.01 ± 0.01
C22:1	0.05 ± 0.01 ^a^	0.04 ± 0.01 ^b^
C22:6	0.04 ± 0.01 ^a^	0.03 ± 0.01 ^b^
∑ UFA	53.50 ± 0.17 ^A^	51.51 ± 0.28 ^B^
PUFA	4.66 ± 0.07 ^A^	4.10 ± 0.02 ^B^
MUFA	48.83 ± 0.22 ^A^	47.41 ± 0.27 ^B^
n-6/n-3 PUFA	9.50 ± 0.29 ^A^	7.79 ± 0.06 ^B^

Note: ^A,B^ means values in the same row with different letters differ significantly at *p* < 0.01; ^a,b^ means values in the same row with different letters differ significantly at *p* < 0.05. FF (indoor feeding farm) group, *n* = 5; and GF (free grazing farm) group, *n* = 5. SEM: standard error of the mean.

**Table 3:** Amino acid composition of LTL(longissimus thoracis et lumborum ) under different rearing conditions in Heigai pigs.

AA (g/100 g)	FF Group	GF Group
Aspartate	1.98 ± 0.02 ^a^	1.79 ± 0.03 ^b^
Threonine	0.76 ± 0.00 ^a^	0.75 ± 0.00 ^b^
Serine	0.75 ± 0.00 ^b^	0.82 ± 0.00 ^a^
Glutamate	2.76 ± 0.02	2.78 ± 0.05
Proline	0.51 ± 0.00 ^b^	0.53 ± 0.01 ^a^
Glycine	0.76 ± 0.01 ^b^	0.78 ± 0.00 ^a^
Alanine	0.99 ± 0.00 ^b^	1.05 ± 0.02 ^a^
Cysteine	0.20 ± 0.00	0.20 ± 0.00
Valine	1.03 ± 0.02	1.05 ± 0.01
Methionine	0.52 ± 0.00 ^b^	0.53 ± 0.00 ^a^
Isoleucine	0.99 ± 0.00	1.00 ± 0.01
Leucine	1.44 ± 0.04	1.40 ± 0.01
Tyrosine	0.85 ± 0.00	0.85 ± 0.00
Phenylalanine	0.81 ± 0.00 ^b^	0.82 ± 0.01 ^a^
Lysine	1.69 ± 0.00	1.67 ± 0.01
Histidine	0.78 ± 0.00 ^b^	0.80 ± 0.01 ^a^
Arginine	1.20 ± 0.05	1.26 ± 0.00
EAA	7.23 ± 0.03	7.24 ± 0.01
Total protein (%)	20.07 ± 0.18	20.07 ± 0.17

Note: ^a,b^ means values in the same row with different letters differ significantly at *p* < 0.05. FF (indoor feeding farm) group, *n* = 5; and GF (free grazing farm) group, *n* = 5. SEM: standard error of the mean.

**Table 4:** Activities of muscle metabolic enzymes in the longissimus thoracis et lumborum (LTL) and psoas major muscle (PMM) under different rearing conditions in Heigai pigs.

Items	FF	GF
LTL		
LDH, U/g of protein	11,184.81 ± 297.83	10,563.8 ± 484.46
MDH, U/mg of protein	1.56 ± 0.17 ^a^	0.92 ± 0.18 ^b^
SDH, U/mg of protein	5.41 ± 0.25 ^a^	3.80 ± 0.32 ^b^
PMM		
LDH, U/g of protein	10,367.02 ± 228.74 ^b^	11,389.73 ± 263.18 ^a^
MDH, U/mg of protein	0.97 ± 0.17	1.10 ± 0.17
SDH, U/mg of protein	4.50 ± 0.45	3.33 ± 0.34

Note: ^a,b^ means values in the same row with different letters differ significantly at *p* < 0.05. ‘LDH’: lactate dehydrogenase, ’SDH’: succinic dehydrogenase, ‘MDH’: malate dehydrogenase FF (indoor feeding farm) group, *n* = 5; and GF (free grazing farm) group, *n* = 5.. SEM: standard error of the mean.

## Data Availability

The data presented in this study are available in the article.
